# Associação da Gravidade da Regurgitação da Valva Pulmonar com Biomarcadores, Capacidade Funcional e Complicações em Pacientes com Insuficiência Cardíaca

**DOI:** 10.36660/abc.20250088

**Published:** 2026-01-09

**Authors:** Fatih Aydin, Bektas Murat, Selda Murat, Muhammet Burak Daghan

**Affiliations:** 1 TC Sağlik Bakanliği Eskişehir Şehir Hastanesi Odunpazari Turquia TC Sağlik Bakanliği Eskişehir Şehir Hastanesi, Odunpazari – Turquia; 2 Eskisehir Osmangazi University Medical Faculty Department of Cardiology Eskisehir Turquia Eskisehir Osmangazi University - Medical Faculty Department of Cardiology, Eskisehir – Turquia

**Keywords:** Insuficiência da Valva Pulmonar, Teste de Caminhada, Insuficiência Cardíaca

## Abstract

**Fundamento:**

A regurgitação da válvula pulmonar (RP) é frequentemente encontrada em doenças cardíacas, incluindo insuficiência cardíaca (IC). Embora geralmente tolerada, a RP grave pode levar à disfunção do ventrículo direito e a desfechos clínicos negativos; no entanto, seu impacto específico na população mais ampla com IC ainda precisa ser melhor elucidado.

**Objetivos:**

Este estudo foi concebido para avaliar a associação entre a gravidade da insuficiência pulmonar e os níveis do pró-peptídeo natriurético tipo B N-terminal (pró-BNP), seu efeito na capacidade funcional medida pelo teste de caminhada de 6 minutos (TC6M) e a incidência de problemas clínicos significativos em pacientes com IC.

**Métodos:**

Entre 2016 e 2023, realizamos um estudo retrospectivo envolvendo 579 pacientes com IC submetidos a ecocardiografia em duas instituições terciárias. Com base na gravidade da RP avaliada de forma semiquantitativa, os pacientes foram classificados em quatro grupos: sem RP, RP leve, RP moderada e RP grave. As comparações entre os grupos foram realizadas utilizando os testes Qui-quadrado e Kruskal-Wallis. Foram realizadas análises de regressão linear multivariada e correlação de Spearman para avaliar as associações.

**Resultados:**

Os níveis de pró-BNP aumentaram significativamente em todos os grupos de gravidade da RP (Mediana: 2.157 pg/mL [Sem RP] a 23.541 pg/mL [RP Grave], p<0,0001). Em contrapartida, a distância percorrida no TC6M diminuiu significativamente com o agravamento da gravidade da RP (Mediana: 254 m [Sem RP] a 72 m [RP Grave], p<0,0001). A prevalência de ortopneia e derrame pleural também aumentou com a gravidade da RP. Após ajuste multivariado, a gravidade da RP permaneceu associada de forma independente a níveis mais elevados de pró-BNP (β=0,48, p=0,002) e a uma menor distância percorrida no TC6M (β=-0,39, p=0,008).

**Conclusão:**

Em pacientes com IC, o aumento da gravidade da RP está associado de forma independente a níveis elevados de pró-BNP, capacidade funcional reduzida e maior incidência de complicações clínicas.

## Graphical abstract

**Figura Central:**
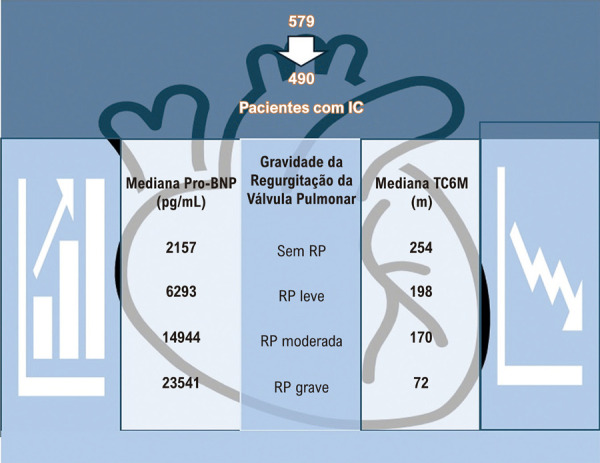
Associação da Gravidade da Regurgitação da Valva Pulmonar com Biomarcadores, Capacidade Funcional e Complicações em Pacientes com Insuficiência Cardíaca

## Introdução

A regurgitação da válvula pulmonar (RP) é um achado clássico em diversas doenças cardíacas, frequentemente observada no contexto de cardiopatia congênita, hipertensão pulmonar e insuficiência cardíaca (IC). Embora a RP leve seja geralmente bem tolerada, a RP grave pode causar sobrecarga de volume e disfunção do ventrículo direito (VD), o que pode levar ao agravamento dos sintomas e desfechos da insuficiência cardíaca.^1-4^

Os efeitos da RP na capacidade funcional e em biomarcadores estabelecidos em populações com IC continuam sendo uma área ativa de pesquisa. O peptídeo natriurético tipo B (BNP) e o pró-BNP N-terminal (pró-BNP) têm sido amplamente pesquisados e servem como biomarcadores-chave para o diagnóstico, prognóstico e monitoramento da IC. Níveis elevados desses peptídeos refletem aumento do estresse da parede ventricular e se correlacionam com eventos clínicos adversos.^5-7^ O teste de caminhada de 6 minutos (TC6M) é uma avaliação padronizada da capacidade funcional em pacientes com IC; ele fornece informações prognósticas relevantes e independentes sobre a tolerância ao exercício e o prognóstico geral.^8,9^

Este estudo teve como objetivo investigar a relação entre a gravidade da insuficiência pulmonar e os níveis de pró-BNP e o TC6M, juntamente com a incidência de ortopneia e derrame pleural nesta coorte de pacientes com IC. Nossa hipótese era de que o aumento da gravidade da RP estaria associado a níveis mais elevados de pró-BNP, redução da capacidade funcional e maior incidência de complicações da IC.

## Métodos

### Desenho do estudo e população de pacientes

Realizamos uma análise retrospectiva de pacientes diagnosticados com IC que realizaram ecocardiografia em dois grandes hospitais terciários entre janeiro de 2016 e dezembro de 2023. Inicialmente, 579 pacientes que atendiam a esses critérios foram identificados. No entanto, para garantir a robustez da análise que exigia variáveis ecocardiográficas e clínicas específicas, a coorte final do estudo incluiu 490 pacientes com conjuntos de dados completos (Figura Central). Os dados foram extraídos de prontuários eletrônicos, abrangendo dados demográficos dos pacientes, histórico médico relevante (incluindo comorbidades como doença arterial coronariana, diabetes, hipertensão e doença pulmonar obstrutiva crônica [DPOC]), resultados de exames laboratoriais padrão, como pró-BNP e hemoglobina, distâncias percorridas no TC6M, quando realizado, e sintomas documentados, como ortopneia ou evidência de derrame pleural. Variáveis conhecidas por influenciar o desempenho no TC6M, incluindo idade, sexo, certas condições clínicas e parâmetros laboratoriais, foram incluídas quando disponíveis. Devido à natureza retrospectiva do estudo, os dados sobre peso e índice de massa corporal dos pacientes não estavam consistentemente disponíveis em toda a coorte. O estudo foi aprovado pelo Comitê de Ética local (Parecer nº: ESH/BAEK 2025/105, datado de: 22/01/2025) e seguiu os princípios da Declaração de Helsinque. Os pacientes foram identificados com base em um diagnóstico documentado de IC em seus prontuários médicos, confirmado por sintomas clínicos, e este estudo foi concebido para avaliar os achados ecocardiográficos. Os mesmos critérios de inclusão foram aplicados de forma consistente em ambos os centros.

Os pacientes foram incluídos se tivessem diagnóstico documentado de IC, confirmado por sintomas clínicos (p. ex., dispneia, ortopneia, fadiga) e evidência ecocardiográfica de fração de ejeção reduzida ou preservada. Foram consideradas as formas sistólica e diastólica da IC. Os critérios de inclusão e a estratégia de busca, baseados em códigos da CID e prontuários clínicos, foram aplicados uniformemente em ambos os centros.

### Avaliação ecocardiográfica

Todos os pacientes incluídos foram submetidos a ecocardiografia transtorácica como parte de sua avaliação clínica. A gravidade da RP foi determinada por meio de uma abordagem semiquantitativa, integrando múltiplos parâmetros Doppler consistentes com diretrizes estabelecidas, como as publicadas pela *American Society of Echocardiography*.^10^ Essa avaliação abrangente considerou achados do Doppler em cores, como a largura do jato regurgitante em relação ao anel pulmonar, sinais de Doppler de onda contínua avaliando a densidade do jato e o perfil de desaceleração, largura da vena contracta, se mensurável com precisão, e a presença ou ausência de fluxo diastólico reverso nos ramos da artéria pulmonar. Com base nessa avaliação integrada, a RP foi classificada e, para fins analíticos, os pacientes foram categorizados em quatro grupos distintos: Sem RP (Grau 0), RP Leve (Grau 1), RP Moderada (Grau 2) e RP Grave (Graus 3-4).

Dois avaliadores independentes realizaram a classificação ecocardiográfica. Embora não tenha sido realizada uma avaliação sistemática da reprodutibilidade interobservador devido ao desenho retrospectivo do estudo, um protocolo semiquantitativo padronizado foi aplicado em ambos os centros, seguindo as recomendações da ASE (*American Society of Echocardiography*).

Além da avaliação da RP, os ecocardiogramas foram revisados para outros parâmetros estruturais e funcionais importantes. A função ventricular esquerda foi avaliada por meio da fração de ejeção do ventrículo esquerdo (FEVE) e do diâmetro diastólico final do ventrículo esquerdo (DDVE). A avaliação do VD incluiu medidas funcionais como a variação fracional da área (VFA-VD) e a excursão sistólica do plano anular tricúspide (TAPSE), juntamente com a avaliação do tamanho utilizando o diâmetro basal do VD. A pressão sistólica da artéria pulmonar (PSAP) foi estimada utilizando o método da velocidade do jato de regurgitação tricúspide. A presença de regurgitação moderada ou grave em outras válvulas (tricúspide, mitral) e doença valvar aórtica significativa (estenose ou regurgitação) também foi documentada.

### Análise estatística

A normalidade da distribuição dos dados para variáveis contínuas foi verificada utilizando o teste de Shapiro-Wilk. Os dados com distribuição normal são apresentados como média ± desvio padrão (DP), enquanto os dados sem distribuição normal são apresentados como mediana [intervalo interquartil, IIQ]. Os dados categóricos são apresentados como contagens e porcentagens (n, %).

Para comparar as características entre os quatro grupos de gravidade da RP, o teste de Kruskal-Wallis foi empregado para variáveis contínuas, dada a distribuição não normal observada para desfechos importantes como pró-BNP e TC6M. Quando diferenças significativas foram encontradas, testes post-hoc de Dunn-Bonferroni foram utilizados para comparações pareadas. As diferenças nas variáveis categóricas entre os grupos foram avaliadas utilizando testes de qui-quadrado ou o teste exato de Fisher, quando apropriado.

Exploramos as relações lineares entre a gravidade da RP (como variável ordinal) e outros parâmetros usando o coeficiente de correlação de Spearman (ρ). Para esta análise, os valores de pró-BNP foram transformados logaritmicamente para melhor aproximar uma distribuição normal.

Além disso, para avaliar se a gravidade da RP estava associada de forma independente aos principais desfechos, realizamos análises de regressão linear multivariada. Modelos separados foram construídos com o pró-BNP e a distância percorrida no TC6M como variáveis dependentes. Ambos os modelos incluíram a gravidade da RP (ordinal), idade, FEVE, PSAP) e hemoglobina como variáveis preditoras independentes. Os coeficientes de regressão padronizados (β) são apresentados.

Todos os testes estatísticos foram bicaudais e um valor p inferior a 0,05 foi considerado estatisticamente significativo. As análises foram realizadas utilizando o SPSS Statistics versão 23.0 (IBM Corp., Armonk, NY, EUA).

## Resultados

### Dados demográficos e características clínicas

O último grupo de estudo consistiu em 490 indivíduos com diagnóstico de IC. As características demográficas e clínicas basais, estratificadas de acordo com a gravidade da RP, estão detalhadas na Tabela 1. Os pacientes nos quatro grupos de RP (sem RP, RP leve, RP moderada e RP grave) apresentaram diferenças significativas na função renal, com taxas de filtração glomerular estimadas (TFG) mais baixas observadas naqueles com RP mais grave. Da mesma forma, os níveis de hemoglobina foram significativamente menores em pacientes com RP moderada ou grave em comparação com aqueles sem RP ou com RP leve. Outros parâmetros laboratoriais, como creatinina e proteína total, também diferiram significativamente entre os grupos. A prevalência de comorbidades importantes, como hipertensão arterial, doença arterial coronariana, diabetes mellitus e DPOC, bem como o uso de medicamentos essenciais para IC, como inibidores da ECA/BRA e betabloqueadores, não apresentou diferenças estatisticamente significativas entre os grupos de gravidade da RP nesta coorte.

**Tabela 1:** – Dados demográficos e características clínicas

Parâmetro	Sem RP (n=272)	RP leve (n=162)	RP moderada (n=20)	RP grave (n=36)	Valor-p^1^
**Idade (anos)**	69,3 ± 10,9	67,2 ± 11,6	72,5 ± 11,2	75,7 ± 12,7	0,198
**Gênero (Masculino/Feminino)**	150/122 (55,1%/44,9%)	90/72 (55,6%/44,4%)	12/8 (60%/40%)	20/16 (55,6%/44,4%)	0,116
**TFG (mL/min/1,73m^2^)**	75,0 ± 15,0	70,0 ± 14,0	65,0 ± 13,0	60,0 ± 12,0	<0,001
**HGB (g/dL)**	12,72 ± 2,01	12,10 ± 1,85	11,80 ± 1,60	11,50 ± 1,42	0,0004
**Cr (mg/dL)**	1,1 ± 0,3	1,2 ± 0,4	1,3 ± 0,5	1,4 ± 0,6	0,0013
**PROTEÍNA TOTAL (g/dL)**	6,5 ± 0,8	6,3 ± 0,7	6,1 ± 0,6	5,9 ± 0,5	0,0022
**PLT (x10^3^/μL)**	220 ± 50	210 ± 45	200 ± 40	190 ± 35	0,0555
**AST (U/L)**	25 ± 10	27 ± 11	29 ± 12	31 ± 13	0,1614
**ALT (U/L)**	30 ± 12	32 ± 13	34 ± 14	36 ± 15	0,2605
**Na (mmol/L)**	140 ± 4	139 ± 4	138 ± 4	137 ± 4	0,3953
**K (mmol/L)**	4,2 ± 0,5	4,3 ± 0,6	4,4 ± 0,7	4,5 ± 0,8	0,4424
**Leucócitos (x10^3^/μL)**	7,5 ± 2,0	7,8 ± 2,1	8,0 ± 2,2	8,2 ± 2,3	0,8344
**Glicose (mg/dL)**	110 ± 20	112 ± 22	115 ± 25	118 ± 28	0,8835
**Hipertensão (%)**	180 (66,2%)	110 (67,9%)	15 (75,0%)	25 (69,4%)	0,192
**DAC (%)**	120 (44,1%)	70 (43,2%)	10 (50,0%)	16 (44,4%)	0,4678
**DM (%)**	90 (33,1%)	50 (30,9%)	7 (35,0%)	12 (33,3%)	0,5843
**DPOC (%)**	40 (14,7%)	25 (15,4%)	4 (20,0%)	6 (16,7%)	0,8708
**Tabagismo (%)**	80 (29,4%)	50 (30,9%)	7 (35,0%)	12 (33,3%)	0,4442
**IECA/BRA (%)**	150 (55,1%)	90 (55,6%)	12 (60,0%)	20 (55,6%)	0,0896
**Betabloqueador (%)**	100 (36,8%)	60 (37,0%)	8 (40,0%)	14 (38,9%)	0,4638
**ARM (%)**	70 (25,7%)	40 (24,7%)	6 (30,0%)	10 (27,8%)	0,7891
**Furosemida (%)**	120 (44,1%)	80 (49,4%)	10 (50,0%)	18 (50,0%)	0,2089
**Tiazida (%)**	50 (18,4%)	30 (18,5%)	5 (25,0%)	8 (22,2%)	0,0442
**Digoxina (%)**	30 (11,0%)	20 (12,3%)	3 (15,0%)	5 (13,9%)	0,5339
**BCC (%)**	60 (22,1%)	40 (24,7%)	5 (25,0%)	8 (22,2%)	0,7220
**Amiodarona (%)**	10 (3,7%)	8 (4,9%)	2 (10,0%)	3 (8,3%)	0,8636
**Ivabradina (%)**	20 (7,4%)	15 (9,3%)	3 (15,0%)	5 (13,9%)	0,4454

TFG: Taxa de Filtração Glomerular; HGB: Hemoglobina; Cr: Creatinina; PLT: Plaquetas; AST: Aspartato Aminotransferase; ALT: Alanina Aminotransferase; Na: Sódio; K: Potássio; WBC: Contagem de Glóbulos Brancos; DAC: Doença Arterial Coronariana; DM: Diabetes Mellitus; DPOC: Doença Pulmonar Obstrutiva Crônica; IECA/BRA: Inibidor da Enzima Conversora de Angiotensina/Bloqueador do Receptor de Angiotensina II; ARM: Antagonista do Receptor de Mineralocorticoides; BCC: Bloqueador dos Canais de Cálcio.

### Resultados ecocardiográficos basais

Uma comparação detalhada dos parâmetros ecocardiográficos basais é apresentada na Tabela 2. Embora a FEVE e o DDVE tenham sido semelhantes entre os grupos de gravidade da RP, diferenças significativas surgiram nos parâmetros do coração direito. A função sistólica do VD, avaliada tanto pela VFA-VD quanto pela TAPSE, demonstrou um declínio significativo com o aumento da gravidade da RP. Consistente com a piora da função do VD, o tamanho do VD, medido pelo diâmetro basal, também aumentou significativamente com maior gravidade da RP. A PSAP estimada mostrou um aumento claro e gradual entre os grupos, sendo significativamente maior em pacientes com RP mais grave. Em relação ao envolvimento de outras válvulas, a prevalência de doença valvar aórtica moderada a grave foi significativamente maior em pacientes com RP mais grave, enquanto diferenças significativas não foram observadas para a prevalência de doença valvar tricúspide ou mitral moderada a grave. A largura da vena contracta apresentou um aumento gradual significativo entre os grupos de gravidade da RP (0,0 ± 0,0 mm sem RP, 2,4 ± 0,5 mm em RP leve, 4,1 ± 0,6 mm em RP moderada e 6,2 ± 0,9 mm em RP grave; p < 0,001), consistente com a classificação baseada em diretrizes.

**Tabela 2:** – Características ecocardiográficas basais

Parâmetro	Sem RP (n=272)	RP leve (n=162)	RP moderada (n=20)	RP grave (n=36)	Valor-p
**Ventrículo esquerdo**					
FEVE (%)	36 ± 10	35 ± 9	34 ± 8	34 ± 7	0,284
DDFVE (mm)	58 ± 7	58 ± 6	59 ± 6	59 ± 5	0,421
**VD**					
VFAVD (%)	45 ± 6	40 ± 5	34 ± 5	30 ± 4	<0,001
TAPSE (mm)	19 ± 3	17 ± 3	15 ± 2	13 ± 2	<0,001
Diâmetro basal do VD (mm)	34 ± 4	37 ± 5	42 ± 6	45 ± 5	<0,001
VC (mm)	0,0 ± 0,0	2,4 ± 0,5	4,1 ± 0,6	6,2 ± 0,9	< 0,001
**Artéria Pulmonar**					
PASP (mmHg)	34 ± 8	42 ± 10	51 ± 11	60 ± 12	<0,001
**Outras doenças valvares (%)**					
RT moderada a grave (%)	52,5%	54,3%	56,2%	57,1%	0,761
DVM moderada a grave (%)	48,9%	49,4%	50,0%	52,8%	0,815
DVA moderada a grave (%)	14,0%	19,1%	30,0%	41,7%	0,003

Os valores são apresentados como média ± desvio padrão ou percentagem (%). FEVE: Fração de Ejeção do Ventrículo Esquerdo; DDFVE: Diâmetro Diastólico Final do Ventrículo Esquerdo; VFAVD: Variação Fracionária da Área do Ventrículo Direito; TAPSE: Excursão Sistólica do Plano Anular da Tricúspide; VD: Ventrículo Direito; PASP: Pressão Sistólica da Artéria Pulmonar; RT: Regurgitação Tricúspide; DVM: Doença da Valva Mitral (Regurgitação ou Estenose); DVA: Doença da Valva Aórtica (Regurgitação ou Estenose). VC: Largura da Vena Contracta.

Além da largura da vena contracta, a largura do jato em relação ao anel pulmonar e os perfis de densidade do jato no Doppler de onda contínua foram qualitativamente consistentes com a classificação de gravidade. No entanto, devido às limitações retrospectivas, os dados quantitativos para densidade do jato e inclinação da desaceleração não puderam ser extraídos de forma confiável e, portanto, não foram apresentados em formato tabular. Mesmo assim, esses parâmetros contribuíram para a abordagem integrada de classificação da insuficiência pulmonar.

### Associação da gravidade da RP com os níveis de pró-BNP

**Figura 1:**
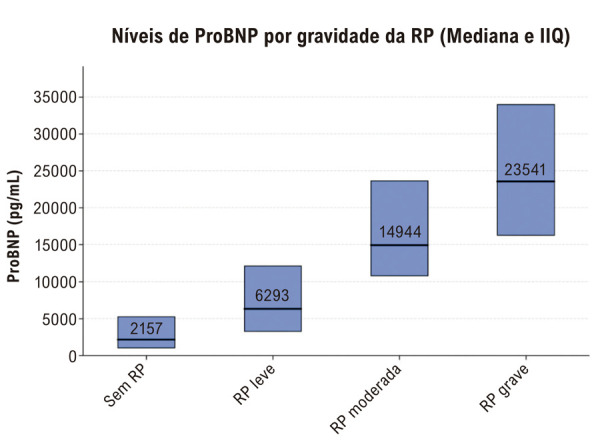
– Níveis de pró-BNP por gravidade da RP.

Os níveis de pró-BNP apresentaram uma forte associação positiva com a gravidade da RP (Tabela 3, Figura 1). Os níveis medianos de pró-BNP aumentaram substancialmente em todos os grupos, com níveis significativamente mais elevados observados em pacientes com RP mais grave (teste de Kruskal-Wallis geral, p < 0,0001).

**Tabela 3:** – Níveis de pró-BNP

Grupo Sem RP	N	Mediana Pro-BNP (pg/mL)	DP	Mediana pró-BNP mediano (pg/mL)	IIQ [Q1, Q3] (pg/mL)	Valor-p
**Sem RP**	272	5.120	4.900	2.157	1.040 – 5.250	
**RP Leve**	162	9.480	7.200	6.293	3.280 – 12.110	
**RP Moderada**	20	17.320	10.100	14.944	10.800 – 23.640	
**RP grave**	36	25.810	13.350	23.541	16.300 – 34.000	
**Classificação geral**	490					<0,0001^1^

^1^Valor de p para comparação geral (teste de Kruskal-Wallis). RP: Regurgitação da válvula pulmonar; N: Número de pacientes; DP: Desvio padrão; IIQ: Intervalo interquartil; Pró-BNP: Peptídeo natriurético pró-BNP-terminal.

### Associação da gravidade da RP com a capacidade funcional

**Figura 2:**
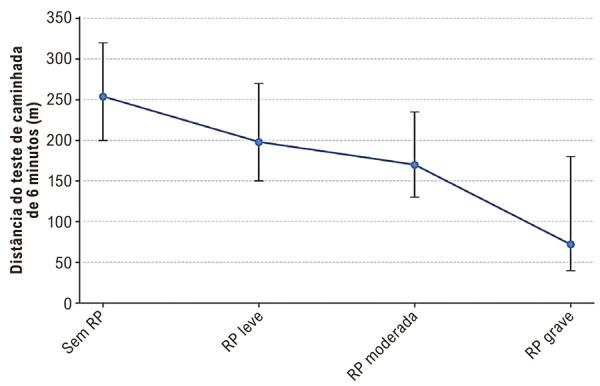
– Distância percorrida no teste de caminhada de 6 minutos por gravidade da RP.

A capacidade funcional, avaliada pelo TC6M, apresentou uma relação inversa significativa com a gravidade da RP (Tabela 4, Figura 2). A distância mediana percorrida diminuiu progressivamente com o agravamento da gravidade da RP, atingindo uma distância significativamente menor no grupo com RP grave em comparação com aqueles sem RP ou com RP leve (teste de Kruskal-Wallis geral, p < 0,0001).

**Tabela 4:** – Análise de regressão multivariada para pró-BNP e TC6M

Variável	β (pró-BNP)	Valor-p	β (6MWT)	Valor-p
Gravidade da RP	0,48	0,002	-0,39	0,008
Idade	0,22	0,041	-0,18	0,058
FEVE	-0,35	0,003	0,29	0,012
PASP	0,41	0,001	-0,33	0,004
Hemoglobina	-0,19	0,038	0,15	0,067

Ajustado por idade, FEVE, PASP e hemoglobina. β: Coeficiente de regressão padronizado. RP: Regurgitação da válvula pulmonar; FEVE: Fração de Ejeção do Ventrículo Esquerdo; PASP: Pressão Sistólica da Artéria Pulmonar.

### Correlações Lineares

**Figura 4:**
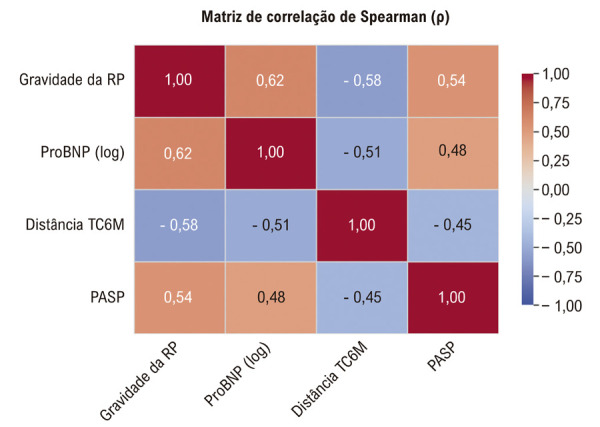
– Matriz de Correlação de Spearman.

Para explorar melhor essas relações, foram realizadas análises de correlação de Spearman (Tabela 5, Figura 4). A gravidade da RP demonstrou uma correlação positiva moderada com os níveis de pró-BNP transformados em logaritmo e uma correlação negativa moderada com a distância percorrida no TC6M. Além disso, a gravidade da RP apresentou correlação significativa com indicadores de alterações hemodinâmicas e estruturais do VD, mostrando correlações positivas com a PSAP e o índice de volume diastólico final do ventrículo direito (VDFVD).

**Tabela 5:** – Distância percorrida no Teste de Caminhada de 6 Minutos (TC6M)

Grupo Sem RP	N	Mediana do 6MWT (m)	DP	Mediana do 6MWT (m)	IIQ [Q1, Q3] (m)	Valor-p
**Sem RP**	272	261,8	104,0	254	200 – 320	
**RP Leve**	162	214,9	87,6	198	150 – 270	
**RP Moderada**	20	187,3	90,4	170	130 – 235	
**RP grave**	36	120,3	83,1	72	40 – 180	
**Classificação geral**	490					<0,0001^1^

^1^Valor de p para comparação geral (teste de Kruskal-Wallis). RP: Regurgitação da válvula pulmonar; N: Número de pacientes; TC6M: Teste de caminhada de 6 minutos; DP: Desvio padrão; IIQ: Intervalo interquartil; m: metros.

### Análise multivariada

Foram realizadas análises de regressão linear multivariada para determinar se a gravidade da RP estava independentemente associada aos níveis de pró-BNP e à distância percorrida no TC6M, após o ajuste para potenciais fatores de confusão (Tabela 5). Controlando para idade, FEVE, PSAP e hemoglobina, a gravidade da RP permaneceu um preditor independente significativo de níveis mais elevados de pró-BNP. Da mesma forma, a gravidade da RP foi independentemente associada a uma menor distância percorrida no TC6M após o ajuste para as mesmas covariáveis. Nesses modelos, a PSAP e a FEVE também emergiram como preditores independentes significativos tanto para o pró-BNP quanto para a distância percorrida no TC6M. Desfechos binários, como ortopneia e derrame pleural, foram avaliados descritivamente entre os grupos; no entanto, modelos de regressão não foram aplicados a essas variáveis devido ao número limitado de eventos e ao risco de sobreajuste do modelo.

O modelo multivariado final explicou 46% da variância nos níveis de pró-BNP (R^2^ = 0,46) e 39% da variância na distância percorrida no TC6M (R^2^ = 0,39), indicando um bom ajuste do modelo.

### Complicações clínicas

**Figura 3:**
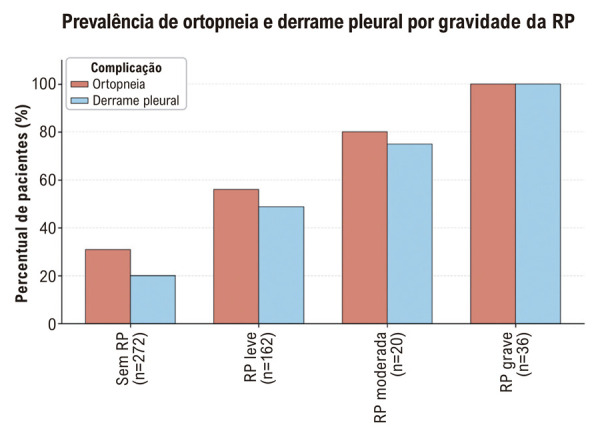
– Prevalência de ortopneia e derrame pleural por gravidade da RP.

A prevalência de ortopneia e derrame pleural aumentou acentuadamente com o avanço da gravidade da RP (Figura 3). Ambas as complicações foram substancialmente mais comuns em pacientes com RP moderada e grave em comparação com aqueles sem RP ou com RP leve. Esses achados destacam a associação significativa entre a gravidade da RP e a carga clínica dos sintomas e complicações da IC.

## Discussão

Este estudo demonstra uma correlação progressiva entre a gravidade da RP e todos os outros parâmetros clínicos em pacientes com IC. O aumento observado nos níveis de pró-BNP com o agravamento da RP pode estar associado ao aumento do estresse da parede ventricular, potencialmente relacionado à sobrecarga de volume do VD frequentemente observada em casos de RP significativa. De forma consistente, nossos achados demonstram um claro aumento gradual nos níveis de pró-BNP com o agravamento da gravidade da RP. Isso está de acordo com outros estudos que mostram que a disfunção do VD leva ao aumento dos níveis de peptídeo natriurético na IC.^11,12^

A diminuição na distância percorrida no TC6M com o aumento da gravidade da RP sugere uma associação entre RP mais grave e capacidade funcional reduzida nessa população. Esse achado está em consonância com pesquisas que enfatizam a importância da função do VD no desempenho físico em IC e é consistente com nossas observações de declínio na fração de área de contração do VD (FAC-VD) e no TAPSE, além do aumento do diâmetro basal do VD com maior gravidade da RP (conforme mostrado na Tabela 2). Isso corrobora ainda mais as pesquisas que enfatizaram o funcionamento do VD no desempenho físico em IC.^8,9-13^

Existem associações entre a gravidade da RP e a presença de ortopneia e derrame pleural, o que nos leva a sugerir que a RP parece contribuir para o desenvolvimento dessas complicações comuns da IC. Os mecanismos fisiopatológicos subjacentes a essa observação parecem ser multifatoriais. A gravidade da RP é responsável por uma sobrecarga significativa em termos de volume e pressão transmitidos ao VD, com consequente dilatação progressiva e comprometimento da contratilidade, além da elevação subsequente das pressões da artéria pulmonar, o que cria um ciclo vicioso de aumento da regurgitação e disfunção do VD.^14-15^ Além disso, a disfunção crônica do VD também pode desempenhar um papel no enchimento do ventrículo esquerdo, utilizando a interdependência ventricular, aumentando assim a sobrecarga hemodinâmica. Os efeitos sistêmicos da RP também implicariam em disfunção biventricular significativa, visto que uma maior carga de trabalho no lado direito do coração causaria alterações no desempenho cardíaco do lado esquerdo.^15-17^

As análises de correlação linear forneceram suporte quantitativo adicional para essas relações (Tabela 6). Correlações moderadas e estatisticamente significativas foram encontradas entre a gravidade da RP e os níveis de pró-BNP transformados em logaritmo, a distância percorrida no TC6M, a PSAP estimada e o índice de volume diastólico final do VD. Essas correlações reforçam a relação entre a gravidade da lesão valvar e parâmetros bioquímicos, funcionais, hemodinâmicos e estruturais adversos em pacientes com IC.

**Tabela 6:** – Coeficientes de correlação (ρ de Spearman)

Variável	Gravidade da RP	Pró-BNP (log)	Distância 6MWT	PASP
Gravidade da RP	1,00	0,62*	−0,58*	0,54*
pró-BNP (log)	0,62*	1,00	−0,51*	0,48*
Distância 6MWT	−0,58*	−0,51*	1,00	−0,45*
PASP (mmHg)	0,54*	0,48*	−0,45*	1,00

*p < 0,001 para todos os coeficientes (bicaudal); os valores de pro-BNP foram transformados logaritmicamente para normalizar a distribuição; RP: Regurgitação da válvula pulmonar; PASP: Pressão sistólica da artéria pulmonar.

Clinicamente, nossos achados reforçam a importância da avaliação rotineira da gravidade da RP em pacientes com IC.^18,19^ A associação independente demonstrada entre a gravidade da RP e os níveis de pró-BNP e a capacidade funcional sugere sua potencial utilidade na estratificação de risco e na orientação das decisões de tratamento. A identificação de pacientes com RP moderada a grave pode destacar um subgrupo que poderia se beneficiar de intervenções direcionadas, embora sejam necessárias mais pesquisas. Portanto, a integração da avaliação da RP na avaliação abrangente de pacientes com IC parece justificada, pois fornece informações prognósticas valiosas que podem influenciar as estratégias terapêuticas.^16^ Dada a associação conhecida entre níveis elevados de pró-BNP e aumento da mortalidade em outros estudos, nossos achados destacam sua potencial utilidade para permitir a estratificação de risco e a tomada de decisões no tratamento.^20,21^ Por exemplo, o tratamento de pacientes com RP moderada a grave em pacientes com IC pode ajudar a mitigar a mortalidade e a morbidade. Este estudo, portanto, fornece implicações clínicas úteis. A avaliação da gravidade da RP deve ser incorporada à avaliação rotineira de pacientes com IC, uma vez que fornece informações prognósticas valiosas que podem influenciar nossa abordagem de tratamento. Portanto, se justifica a realização de mais pesquisas sobre a eficácia potencial de intervenções para redução da pressão arterial, como a substituição da válvula pulmonar ou intervenções transcateter, na melhoria dos resultados em pacientes com IC.

### Limitações

Este estudo apresenta diversas limitações que devem ser reconhecidas. Em primeiro lugar, seu desenho retrospectivo inerentemente acarreta riscos de viés de seleção e depende de dados coletados para fins clínicos, que podem não ser uniformemente detalhados em todos os pacientes. Em segundo lugar, a avaliação retrospectiva da gravidade da RP é uma limitação importante. Embora nossa classificação tenha utilizado uma abordagem abrangente alinhada às diretrizes, nem todos os parâmetros Doppler puderam ser extraídos de forma confiável dos prontuários clínicos. Validamos nossa classificação demonstrando que ela era corroborada pelos dados disponíveis, especialmente a largura da vena contracta, que aumentou com o agravamento da gravidade da RP. No entanto, a ausência de dados quantitativos completos significa que estudos prospectivos futuros com exames de imagem padronizados são necessários para confirmar esses achados. Em terceiro lugar, o estudo foi conduzido em dois centros, o que pode limitar a generalização dos resultados para outras populações ou contextos de saúde. Além disso, o número relativamente pequeno de pacientes nos grupos com RP moderada e grave pode ter limitado o poder estatístico para análises de subgrupos.

Embora nossa análise multivariada tenha identificado a gravidade da RP como um preditor independente de pró-BNP e da distância percorrida no TC6M após o ajuste para diversos fatores de confusão importantes, a causalidade não pode ser definitivamente estabelecida a partir deste estudo observacional. Apesar de termos ajustado para fatores de confusão importantes como idade, FEVE, PSAP e hemoglobina, nosso desenho retrospectivo impediu a coleta consistente de dados sobre peso e índice de massa corporal dos pacientes. Essa limitação significa que fatores não mensurados podem ter influenciado as diferenças observadas na distância percorrida no TC6M entre os grupos de gravidade da RP. Pode haver confusão residual devido a variáveis não mensuradas ou complexidades na interação entre RP, disfunção do VD e progressão geral da IC que não foram totalmente capturadas. Embora tenhamos incluído preditores importantes como FEVE e PSAP, os dados retrospectivos limitaram nossa capacidade de incluir outros fatores potencialmente importantes de forma uniforme em toda a coorte. Portanto, os achados devem ser interpretados como demonstrando fortes associações independentes, e não causalidade direta. Estudos prospectivos com coleta de dados abrangente e metodologias potencialmente avançadas são necessários para confirmar esses achados e elucidar ainda mais as vias causais.

## Conclusão

Em conclusão, este estudo demonstra que o aumento da gravidade da insuficiência pulmonar está associado de forma independente a níveis elevados de pró-BNP, redução da capacidade funcional medida pelo TC6M e maior frequência de complicações como ortopneia e derrame pleural em pacientes com insuficiência cardíaca. Esses achados destacam a importância clínica da avaliação da gravidade da insuficiência pulmonar na avaliação abrangente, estratificação de risco e possível manejo de pacientes com insuficiência cardíaca.
