# Supplementary material for: Associação da Gravidade da Regurgitação da Valva Pulmonar com Biomarcadores, Capacidade Funcional e Complicações em Pacientes com Insuficiência Cardíaca
Source: Arq Bras Cardiol. 2026 Jan 9;122(12):e20250088. [Article in Portuguese] doi: 10.36660/abc.20250088 (PMC12978364; doi:10.36660/abc.20250088)
Supplement: Translation

# Translation: Association of the Severity of Pulmonary Valve Regurgitation on Biomarkers, Functional Capacity, and Complications in Patients with Heart Failure

**DOI:** 10.36660/abc.20250088i

**Authors:** Fatih Aydin, Bektas Murat, Selda Murat, Muhammet Burak Daghan

**Affiliations:** 1 Türkiye TC Sağlik Bakanliği Eskişehir Şehir Hastanesi, Odunpazari – Türkiye; 2 Türkiye Eskisehir Osmangazi University - Medical Faculty Department of Cardiology, Eskisehir – Türkiye

**Keywords:** Pulmonary Valve Insufficiency, Walk Test, Heart Failure

## Abstract

**Background:**

Pulmonary valve regurgitation (PR) is often encountered in cardiac conditions, including heart failure (HF). Although typically tolerated, severe PR can lead to right ventricular dysfunction and negative clinical outcomes; however, its specific impact within the wider HF population needs further clarification.

**Objectives:**

This study was designed to evaluate the association between the severity of PR and N-terminal pro-B-type natriuretic peptide (pro-BNP) levels, its effect on functional capacity measured by the six-minute walk test (6MWT), and the incidence of significant clinical problems in patients with HF.

**Methods:**

Between 2016 and 2023, we conducted a retrospective study involving 579 HF patients who underwent echocardiography at two tertiary institutions. Based on semi-quantitatively evaluated PR severity, patients were classified into four groups: No PR, Mild PR, Moderate PR, and Severe PR. Group comparisons used Chi-square tests and Kruskal-Wallis. Multivariate linear regression and Spearman correlation analyses were performed to assess associations.

**Results:**

Pro-BNP levels significantly increased across PR severity groups (Median: 2,157 pg/mL [No PR] to 23,541 pg/mL [Severe PR], p<0.0001). In contrast, 6MWT distance significantly decreased with deteriorating PR severity (Median: 254 m [No PR] to 72 m [Severe PR], p<0.0001). The prevalence of orthopnea and pleural effusion also increased with PR severity. After multivariate adjustment, PR severity remained independently associated with higher pro-BNP levels (β=0.48, p=0.002) and lower 6MWT distance (β=-0.39, p=0.008).

**Conclusion:**

In patients with HF, increasing severity of PR is independently associated with elevated pro-BNP levels, reduced functional capacity, and a higher burden of clinical complications.

## Introduction

Pulmonary valve regurgitation (PR) is a classic finding in various cardiac diseases, often noted in the context of congenital heart disease, pulmonary hypertension, and heart failure (HF). While mild PR is generally well tolerated, severe PR can cause right ventricular (RV) volume overload and dysfunction that may ultimately lead to worsening HF symptoms and outcomes.^1-4^

The effects of PR on functional capacity and established biomarkers in HF populations remain an active area of research. B-type natriuretic peptide (BNP) and N-terminal pro-BNP (pro-BNP) have been widely researched and serve as key biomarkers for diagnosing, prognosticating, and monitoring HF. High levels of these peptides reflect increased ventricular wall stress and correlate with adverse clinical events.^5-7^ The six-minute walk test (6MWT) is a standardized assessment of functional capacity in HF patients; it provides relevant and independent prognostic information about exercise tolerance and overall prognosis.^8,9^

This study aimed to investigate the relationship between the severity of PR and levels of Pro-BNP and 6MWT, together with the incidence of orthopnea and pleural effusion in this cohort of HF patients. We hypothesized that the escalating severity of PR was associated with higher pro-BNP levels, reduced functional capacity, and increased burdens of HF complications.

## Methods

### Study Design and Patient Population

We conducted a retrospective analysis of patients diagnosed with HF who underwent echocardiography at two large tertiary care hospitals between January 2016 and December 2023. Initially, 579 patients meeting these criteria were identified. However, to ensure the robustness of the analysis requiring specific echocardiographic and clinical variables, the final study cohort included 490 patients with complete datasets (Central Illustration). Data were extracted from electronic health records, encompassing patient demographics, relevant medical history (including comorbidities like coronary artery disease, diabetes, hypertension, and chronic obstructive pulmonary disease [COPD]), standard laboratory results such as N-terminal pro-B-type natriuretic peptide (pro-BNP), and hemoglobin, 6MWT distances when performed, and documented symptoms like orthopnea or evidence of pleural effusion. Variables known to influence 6MWT performance, including age, gender, certain clinical conditions, and laboratory parameters, were included if available. Due to the retrospective nature of the study, data on patient weight and body mass index were not consistently available across the cohort. The study received approval from the local Ethics Committee (Approval No: ESH/BAEK 2025/105, Dated: 22/01/2025) and adhered to the principles outlined in the Declaration of Helsinki. Patients were identified based on a documented HF diagnosis in the medical records, confirmed by clinical symptoms, and this study was designed to evaluate echocardiographic findings. The same inclusion criteria were applied consistently across both centers.

Patients were included if they had a documented diagnosis of HF confirmed by clinical symptoms (e.g., dyspnea, orthopnea, fatigue) and echocardiographic evidence of reduced or preserved ejection fraction. Both systolic and diastolic forms of HF were considered. Inclusion criteria and search strategy based oncodes and clinical notes were applied uniformly across both centers.

### Echocardiographic Evaluation

All included patients had undergone transthoracic echocardiography as part of their clinical assessment. The severity of PR was determined through a semi-quantitative approach, integrating multiple Doppler parameters consistent with established guidelines, such as those published by the American Society of Echocardiography.^10^ This comprehensive assessment considered color Doppler findings like the regurgitant jet width relative to the pulmonary annulus, continuous-wave Doppler signals evaluating jet density and deceleration profile, vena contracta width if accurately measurable, and the presence or absence of diastolic flow reversal in the pulmonary artery branches. Based on this integrated evaluation, PR was graded, and for analytical purposes, patients were categorized into four distinct groups: No PR (Grade 0), Mild PR (Grade 1), Moderate PR (Grade 2), and Severe PR (Grades 3-4).

Two independent readers performed echocardiographic grading. While systematic interobserver reproducibility assessment was not conducted due to the retrospective design, a standardized semi-quantitative protocol was applied at both centers following ASE (*American Society of Echocardiography*) recommendations.

Beyond the PR assessment, the echocardiograms were reviewed for other key structural and functional parameters. Left ventricular function was assessed using left ventricular ejection fraction (LVEF) and end-diastolic diameter (LVEDD). The RV evaluation included functional measures like right fractional area change (RV-FAC) and tricuspid annular plane systolic excursion (TAPSE), along with size assessment using the RV basal diameter. Pulmonary artery systolic pressure (PASP) was estimated using the tricuspid regurgitation jet velocity method. The presence of moderate or severe regurgitation in other valves (tricuspid, mitral) and significant aortic valve disease (stenosis or regurgitation) was also documented.

### Statistical analysis

Data distribution normality for continuous variables was checked using the Shapiro-Wilk test. Normally distributed data are presented as mean ± standard deviation (SD), while non-normally distributed data are shown as median [Interquartile Range, IQR]. Categorical data are reported as counts and percentages (n, %).

To compare characteristics across the four PR severity groups, the Kruskal-Wallis test was employed for continuous variables, given the non-normal distribution observed for key outcomes like pro-BNP and 6MWT. Where significant differences were found, Dunn-Bonferroni post-hoc tests were used for pairwise comparisons. Differences in categorical variables among the groups were assessed using chi-square tests or Fisher’s exact test when appropriate.

We explored the linear relationships between PR severity (as an ordinal variable) and other parameters using Spearman’s rank correlation coefficient (ρ). For this analysis, pro-BNP values were log-transformed to better approximate a normal distribution.

Furthermore, to assess whether PR severity was independently associated with key outcomes, we performed multivariate linear regression analyses. Separate models were constructed with pro-BNP and 6MWT distance as the dependent variables. Both models included PR severity (ordinal), age, LVEF, PASP, and hemoglobin as independent predictor variables. Standardized regression coefficients (β) are reported.

All statistical tests were two-tailed, and a p-value less than 0.05 was considered statistically significant. Analyses were performed using SPSS Statistics Version 23.0 (IBM Corp., Armonk, NY, USA).

## Results

### Demographics and clinical characteristics

The last study group consisted of 490 HF-diagnosed individuals. Baseline demographic and clinical characteristics, stratified according to the severity of PR, are detailed in Table 1. Patients across the four PR groups (No PR, Mild PR, Moderate PR, Severe PR) showed significant differences in renal function, with lower estimated glomerular filtration rates (GFR) observed in those with more severe PR. Similarly, hemoglobin levels were significantly lower in patients with moderate or severe PR compared to those with no or mild PR. Other laboratory parameters, like creatinine and total protein, also differed significantly across the groups. The prevalence of major comorbidities such as hypertension, coronary artery disease, diabetes mellitus, and COPD, as well as the use of key HF medications like ACE inhibitors/ARBs and beta-blockers, did not show statistically significant differences among the PR severity groups in this cohort.

**Table 1:** – Demographics and Clinical Characteristics

Parameter	No PR (n=272)	Mild PR (n=162)	Moderate PR (n=20)	Severe PR (n=36)	p-value^1^
**Age (years)**	69.3 ± 10.9	67.2 ± 11.6	72.5 ± 11.2	75.7 ± 12.7	0.198
**Gender (Male/Female)**	150/122 (55.1%/44.9%)	90/72 (55.6%/44.4%)	12/8 (60%/40%)	20/16 (55.6%/44.4%)	0.116
**GFR (mL/min/1.73m^2^)**	75.0 ± 15.0	70.0 ± 14.0	65.0 ± 13.0	60.0 ± 12.0	<0.001
**HGB (g/dL)**	12.72 ± 2.01	12.10 ± 1.85	11.80 ± 1.60	11.50 ± 1.42	0.0004
**Cr (mg/dL)**	1.1 ± 0.3	1.2 ± 0.4	1.3 ± 0.5	1.4 ± 0.6	0.0013
**TPROTEIN (g/dL)**	6.5 ± 0.8	6.3 ± 0.7	6.1 ± 0.6	5.9 ± 0.5	0.0022
**PLT (x10^3^/μL)**	220 ± 50	210 ± 45	200 ± 40	190 ± 35	0.0555
**AST (U/L)**	25 ± 10	27 ± 11	29 ± 12	31 ± 13	0.1614
**ALT (U/L)**	30 ± 12	32 ± 13	34 ± 14	36 ± 15	0.2605
**Na (mmol/L)**	140 ± 4	139 ± 4	138 ± 4	137 ± 4	0.3953
**K (mmol/L)**	4.2 ± 0.5	4.3 ± 0.6	4.4 ± 0.7	4.5 ± 0.8	0.4424
**WBC (x10^3^/μL)**	7.5 ± 2.0	7.8 ± 2.1	8.0 ± 2.2	8.2 ± 2.3	0.8344
**Glucose (mg/dL)**	110 ± 20	112 ± 22	115 ± 25	118 ± 28	0.8835
**Hypertension (%)**	180 (66.2%)	110 (67.9%)	15 (75.0%)	25 (69.4%)	0.192
**CAD (%)**	120 (44.1%)	70 (43.2%)	10 (50.0%)	16 (44.4%)	0.4678
**DM (%)**	90 (33.1%)	50 (30.9%)	7 (35.0%)	12 (33.3%)	0.5843
**COPD (%)**	40 (14.7%)	25 (15.4%)	4 (20.0%)	6 (16.7%)	0.8708
**Smoking (%)**	80 (29.4%)	50 (30.9%)	7 (35.0%)	12 (33.3%)	0.4442
**ACEI/ARB (%)**	150 (55.1%)	90 (55.6%)	12 (60.0%)	20 (55.6%)	0.0896
**Beta-blocker (%)**	100 (36.8%)	60 (37.0%)	8 (40.0%)	14 (38.9%)	0.4638
**MRA (%)**	70 (25.7%)	40 (24.7%)	6 (30.0%)	10 (27.8%)	0.7891
**Furosemide (%)**	120 (44.1%)	80 (49.4%)	10 (50.0%)	18 (50.0%)	0.2089
**Tiazid (%)**	50 (18.4%)	30 (18.5%)	5 (25.0%)	8 (22.2%)	0.0442
**Digoxin (%)**	30 (11.0%)	20 (12.3%)	3 (15.0%)	5 (13.9%)	0.5339
**CCB (%)**	60 (22.1%)	40 (24.7%)	5 (25.0%)	8 (22.2%)	0.7220
**Amiodarone (%)**	10 (3.7%)	8 (4.9%)	2 (10.0%)	3 (8.3%)	0.8636
**Ivabradine (%)**	20 (7.4%)	15 (9.3%)	3 (15.0%)	5 (13.9%)	0.4454

GFR: Glomerular Filtration Rate; HGB: Hemoglobin; Cr: Creatinine; TPROTEIN: Total Protein; PLT: Platelet; AST: Aspartate Aminotransferase; ALT: Alanine Aminotransferase; Na: Sodium; K: Potassium; WBC: White Blood Cell Count; CAD: Coronary Artery Disease; DM: Diabetes Mellitus; COPD: Chronic Obstructive Pulmonary Disease; ACEI/ARB: Angiotensin-Converting Enzyme Inhibitor/Angiotensin II Receptor Blocker; MRA: Mineralocorticoid Receptor Antagonist; CCB: Calcium Channel Blocker.

### Baseline echocardiographic findings

A detailed comparison of baseline echocardiographic parameters is presented in Table 2. While LVEF and LVEDD were similar across the PR severity groups, significant differences emerged in right heart parameters. RV systolic function, assessed by both RV-FAC and TAPSE, demonstrated a significant decline with increasing PR severity. Consistent with worsening RV function, RV size measured by basal diameter also increased significantly with greater PR severity. Estimated PASP showed a clear, graded increase across the groups, being significantly higher in patients with more severe PR. Regarding other valve involvement, the prevalence of moderate-to-severe aortic valve disease was significantly higher in patients with more severe PR, whereas significant differences were not observed for moderate-to-severe tricuspid or mitral valve disease prevalence. Vena contracta width showed a significant stepwise increase across PR severity groups (0.0 ± 0.0 mm in no PR, 2.4 ± 0.5 mm in mild, 4.1 ± 0.6 mm in moderate, and 6.2 ± 0.9 mm in severe PR; p < 0.001), consistent with guideline-based classification.

**Table 2:** – Baseline Echocardiographic Characteristics

Parameter	No PR (n=272)	Mild PR (n=162)	Moderate PR (n=20)	Severe PR (n=36)	p-value
**Left Ventricle**					
LVEF (%)	36 ± 10	35 ± 9	34 ± 8	34 ± 7	0.284
LVEDD (mm)	58 ± 7	58 ± 6	59 ± 6	59 ± 5	0.421
**Right Ventricle**					
RV-FAC (%)	45 ± 6	40 ± 5	34 ± 5	30 ± 4	<0.001
TAPSE (mm)	19 ± 3	17 ± 3	15 ± 2	13 ± 2	<0.001
RV basal diameter (mm)	34 ± 4	37 ± 5	42 ± 6	45 ± 5	<0.001
VC (mm)	0.0 ± 0.0	2.4 ± 0.5	4.1 ± 0.6	6.2 ± 0.9	< 0.001
**Pulmonary Artery**					
PASP (mmHg)	34 ± 8	42 ± 10	51 ± 11	60 ± 12	<0.001
**Other Valve Disease (%)**					
Moderate–Severe TR (%)	52.5%	54.3%	56.2%	57.1%	0.761
Moderate–Severe MVD (%)	48.9%	49.4%	50.0%	52.8%	0.815
Moderate–Severe AVD (%)	14.0%	19.1%	30.0%	41.7%	0.003

Values are mean ± SD or percentage (%). LVEF: Left Ventricular Ejection Fraction; LVEDD: Left Ventricular End-Diastolic Diameter; RV-FAC: Right Ventricular Fractional Area Change; TAPSE: Tricuspid Annular Plane Systolic Excursion; RV: Right Ventricle; PASP: Pulmonary Artery Systolic Pressure; TR: Tricuspid Regurgitation; MVD: Mitral Valve Disease (Regurgitation or Stenosis; AVD: Aortic Valve Disease (Regurgitation or Stenosis). VC: Vena Contracta Width.

In addition to vena contracta width, jet width relative to the pulmonary annulus, and continuous-wave Doppler jet density profiles were qualitatively consistent with the severity classification. However, due to retrospective limitations, quantitative data for jet density and deceleration slope could not be extracted reliably and were therefore not presented in tabular form. Nonetheless, these parameters contributed to the integrated PR grading approach.

### Association of PR severity with pro-BNP levels

N-terminal pro-BNP levels exhibited a strong positive association with PR severity (Table 3, Figure 1). Median pro-BNP levels rose substantially across the groups, with significantly higher levels observed in patients with more severe PR (Overall Kruskal-Wallis p < 0.0001).

**Table 3:** – Pro-BNP Levels

PR Group	N	Mean pro-BNP (pg/mL)	SD	Median pro-BNP (pg/mL)	IQR [Q1, Q3] (pg/mL)	p-value
**No PR**	272	5,120	4,900	2,157	1,040 – 5,250	
**Mild PR**	162	9,480	7,200	6,293	3,280 – 12,110	
**Moderate PR**	20	17,320	10,100	14,944	10,800 – 23,640	
**Severe PR**	36	25,810	13,350	23,541	16,300 – 34,000	
**Overall Comp.**	490					<0.0001^1^

^1^p-value for overall comparison (Kruskal-Wallis test). PR: Pulmonary Valve Regurgitation; N: Number of patients; SD: Standard Deviation; IQR: Interquartile Range; Pro-BNP: N-terminal pro-B-type natriuretic peptide.

**Figure 1:** – Pro-BNP Levels by PVR Severity.

### Association of PR severity with functional capacity

Functional capacity, evaluated using the 6MWT, showed a significant inverse relationship with PR severity (Table 4, Figure 2). Median walking distance progressively decreased with worsening PR severity, reaching a significantly lower distance in the severe PR group compared to those with no or mild PR (Overall Kruskal-Wallis p < 0.0001).

**Table 4:** – Multivariate Regression Analysis for pro-BNP and 6MWT

Variable	β (pro-BNP)	p-value	β (6MWT)	p-value
PR Severity	0.48	0.002	-0.39	0.008
Age	0.22	0.041	-0.18	0.058
LVEF	-0.35	0.003	0.29	0.012
PASP	0.41	0.001	-0.33	0.004
Hemoglobin	-0.19	0.038	0.15	0.067

Adjusted for age, LVEF, PASP, and hemoglobin. β: Standardized regression coefficient.

**Figure 2:** – Six-Minute Walk Test Distance by PVR Severity.

### Linear correlations

To further explore these relationships, Spearman correlation analyses were performed (Table 5, Figure 4). PR severity demonstrated a moderate positive correlation with log-transformed pro-BNP levels and a moderate negative correlation with 6MWT distance. Furthermore, PR severity was significantly correlated with indicators of hemodynamic and structural RV changes, showing positive correlations with PASP and RV end-diastolic volume index (RVEDV).

**Table 5:** – Six-Minute Walk Test (6MWT) Distance

PR Group	N	Mean pro-BNP (m)	SD	Median pro-BNP (m)	IQR [Q1, Q3] (m)	p-value
**No PR**	272	261.8	104.0	254	200 – 320	
**Mild PR**	162	214.9	87.6	198	150 – 270	
**Moderate PR**	20	187.3	90.4	170	130 – 235	
**Severe PR**	36	120.3	83.1	72	40 – 180	
**Overall Comp.**	490					<0.0001^1^

^1^p-value for overall comparison (Kruskal-Wallis test). PR: Pulmonary Valve Regurgitation; N: Number of patients; 6MWT: Six-Minute Walk Test; SD: Standard Deviation; IQR: Interquartile Range; m: meters.

**Figure 4:** – Spearman Correlation Matrix.

### Multivariate analysis

Multivariate linear regression analyses were conducted to determine if PR severity was independently associated with pro-BNP and 6MWT distance after accounting for potential confounders (Table 5). Controlling for age, LVEF, PASP, and hemoglobin, PR severity remained a significant independent predictor of higher pro-BNP levels. Similarly, PR severity was independently associated with lower 6MWT distance after adjusting for the same covariates. In these models, PASP and LVEF also emerged as significant independent predictors for both pro-BNP and 6MWT. Binary outcomes such as orthopnea and pleural effusion were evaluated descriptively across groups; however, regression models were not applied to these variables due to the limited number of events and the risk of model overfitting.

The final multivariate model explained 46% of the variance in pro-BNP levels (R^2^ = 0.46) and 39% of the variance in 6MWT distance (R^2^ = 0.39), indicating a good model fit.

### Clinical complications

The prevalence of orthopnea and pleural effusion increased markedly with advancing PR severity (Figure 3). Both complications were substantially more common in patients with moderate and severe PR compared to those with no or mild PR. These findings highlight the significant association between the severity of PR and the clinical burden of HF symptoms and complications.

**Figure 3:** – Prevalence of Orthopnea and Pleural Effusion by PVR Severity.

## Discussion

This study shows a progressive correlation between PR severity and all other clinical parameters in HF patients. The observed increase in pro-BNP levels with worsening PR may be associated with increased ventricular wall stress, potentially related to the RV volume overload often seen with significant pulmonary regurgitation. Consistent with this, our findings demonstrate a clear graded increase in pro-BNP levels with escalating severity of PR. This agrees with other studies showing that RV dysfunction leads to increased levels of natriuretic peptide in HF.^11,12^

The decrease in 6MWT distance with increased severity of PR suggests an association between more severe PR and reduced functional capacity in this population. This finding aligns with research emphasizing the importance of RV function in exercise performance in HF and is consistent with our observations of declining RV-FAC and TAPSE and increasing RV basal diameter with greater PR severity (as shown in Table 2). This further substantiates the research that emphasized the functioning of RV in exercise performance in HF. ^8,9-13^

There exist associations between PR severity and the presence of orthopnea and pleural effusion, leading us to suggest that PR appears to contribute to the development of these common HF complications. The pathophysiological mechanisms underlying this observation appear multifactorial. The severity of PR is responsible for impressive overload in terms of volume and pressure transmitted to RV, with resulting progressive dilation and impairment in contractility, with subsequent elevation of pulmonary artery pressures that create a vicious cycle of increasing regurgitation and RV dysfunction.^14-15^ Moreover, chronic RV dysfunction can also play a role in left ventricular filling utilizing ventricular interdependence, thereby making the hemodynamic burden greater. The systemic effects of PR would also imply much biventricular dysfunction, in that a higher workload on the right side of the heart would cause changes in left-sided cardiac performance.^15-17^

The linear correlation analyses provided further quantitative support for these relationships (Table 6). Moderate, statistically significant correlations were found between PR severity and log-transformed pro-BNP levels, 6MWT distance, estimated PASP, and RV end-diastolic volume index. These correlations reinforce the links between the severity of the valvular lesion and adverse biochemical, functional, hemodynamic, and structural parameters in patients with HF.

**Table 6:** – Correlation Coefficients (Spearman’s ρ)

Variable	PR Severity	Pro-BNP (log)	6MWT Distance	PASP
PR Severity	1.00	0.62*	−0.58*	0.54*
pro-BNP (log)	0.62*	1.00	−0.51*	0.48*
6MWT Distance	−0.58*	−0.51*	1.00	−0.45*
PASP (mmHg)	0.54*	0.48*	−0.45*	1.00

*p < 0.001 for all coefficients (two-tailed); pro-BNP values were log-transformed to normalize distribution; PR: Pulmonary Valve Regurgitation; PASP: Pulmonary artery systolic pressure.

Clinically, our findings underscore the importance of routinely assessing PR severity in patients with HF.^18,19^ The demonstrated independent association between PR severity and both pro-BNP levels and functional capacity suggests its potential utility in risk stratification and guiding management decisions. Identifying patients with moderate to severe PR might highlight a subgroup that could potentially benefit from targeted interventions, although further research is needed. Therefore, integrating PR assessment into the comprehensive evaluation of HF patients seems warranted, as it provides valuable prognostic information that could influence therapeutic strategies.^16^Given the known association between elevated pro-BNP and increased mortality in other studies, our findings highlight their potential usefulness in enabling risk stratification and decision-making in management.^20,21^ For example, treatment of patients with moderate to severe PR in HF patients may help mitigate mortality and morbidity. This study, therefore, provides useful clinical implications. The assessment of PR severity should be incorporated into the routine evaluation of HF patients since it provides valuable prognostic information that could influence our treatment approach. Further research into the potential effectiveness of PR-lowering interventions like pulmonary valve replacement or transcatheter interventions in improving outcomes in HF is therefore warranted.

### Limitations

This study has several limitations that should be acknowledged. Firstly, its retrospective design inherently carries risks of selection bias and relies on data collected for clinical purposes, which may not be uniformly detailed across all patients. Secondly, the retrospective assessment of PR severity is a key limitation. Although our grading used a comprehensive approach aligned with guidelines, not all Doppler parameters could be reliably extracted from clinical records. We validated our classification by showing it was supported by available data, especially vena contracta width, which increased with worsening PR severity. Nonetheless, the missing complete quantitative data means future prospective studies with standardized imaging are needed to confirm these findings. Thirdly, the study was conducted at two centers, which might limit the generalizability of the findings to other populations or healthcare settings. Moreover, the relatively small number of patients in the moderate and severe PR groups may have limited statistical power for subgroup analyses.

Although our multivariate analysis identified PR severity as an independent predictor of pro-BNP and 6MWT distance after adjusting for several key confounders, causality cannot be definitively established from this observational study design. While we adjusted for key confounders like age, LVEF, PASP, and hemoglobin, our retrospective design prevented the consistent collection of data on patient weight and body mass index. This limitation means unmeasured factors could have influenced the observed differences in 6MWT distance across PR severity groups. There may be residual confounding from unmeasured variables or complexities in the interplay between PR, RV dysfunction, and overall HF progression that were not fully captured. Although we included major predictors like LVEF and PASP, the retrospective data limited our ability to include other potentially important factors uniformly across the cohort. Therefore, the findings should be interpreted as demonstrating strong independent associations rather than direct causation. Prospective studies with comprehensive data collection and potentially advanced methodologies are needed to confirm these findings and further elucidate the causal pathways.

## Conclusion

In conclusion, this study demonstrates that increasing severity of PR is independently associated with elevated pro-BNP levels, reduced functional capacity measured by the 6MWT, and a higher frequency of complications such as orthopnea and pleural effusion in patients with HF. These findings highlight the clinical importance of evaluating PR severity in comprehensive assessment, risk stratification, and potential management of patients with HF.
